# Supplementary material for: Celcomen: spatial causal disentanglement for single-cell and tissue perturbation modeling
Source: Nat Commun. 2026 Mar 18;17:4126. doi: 10.1038/s41467-026-69856-5 (PMC13149852; doi:10.1038/s41467-026-69856-5)
Supplement: Supplementary file 1 — Supplementary Information [file 41467_2026_69856_MOESM1_ESM.pdf]

Supplementary Figures

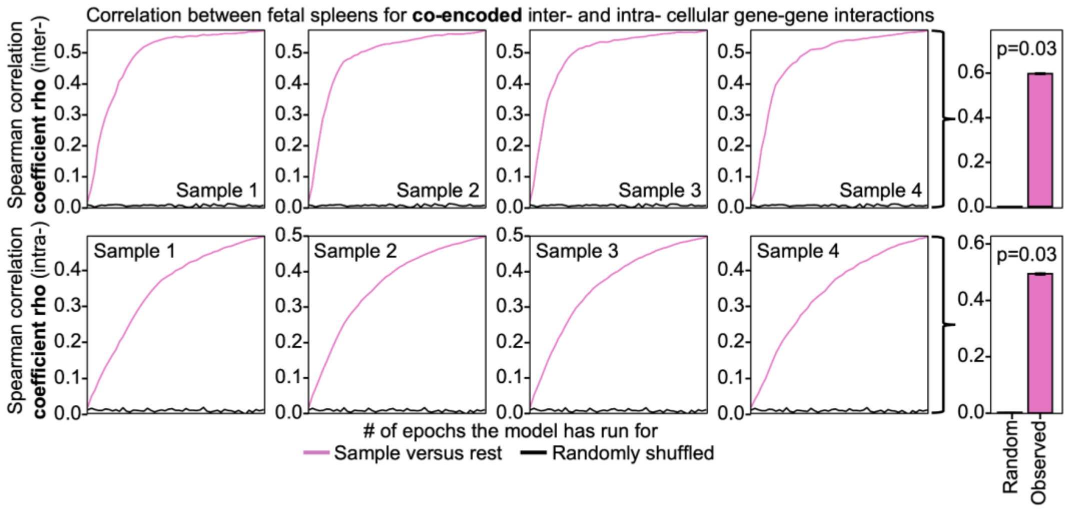

Supplementary Fig. 1 – Celcomen recapitulates its identifiability guarantees through strong sample-to-sample correlation on real human samples: Left: Line plots with the x-axis as epochs and y-axis as the Spearman correlation coefficients between the gene-gene interaction matrices of the model trained on the specified sample and the model trained on all other samples. The sample utilized for the sample specific model is annotated directly on the plot. The color of the line, see lower legend, indicates whether it represents comparisons between the two observed models, pink, or between a random shuffling of the two gene-gene interactions, black, to represent a null model. Right: Bar plots with the left black bar representing the average final Spearman correlation coefficient between randomly shuffled gene-gene interaction matrices of the sample specific model and model trained on all other samples, and the right pink bar representing the observed correlation. P-values are derived from Mann-Whitney U test and are annotated directly on plot. Error bars represent standard error and bar heights represent mean. Source data are provided as a Source Data file.

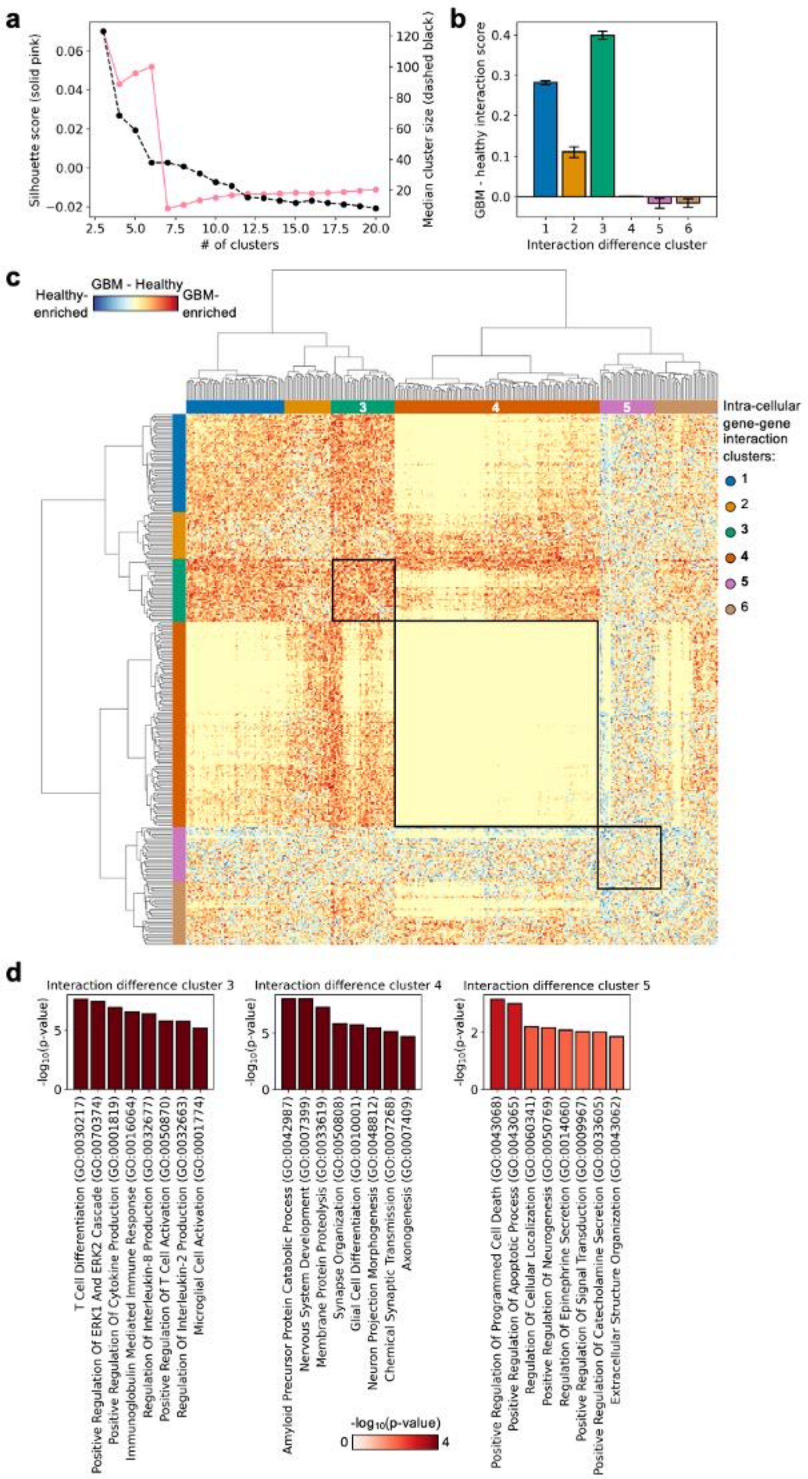

Supplementary Fig. 2 – comparison of intra-cellular gene-gene interactions between glioblastoma and healthy human brains:

a) Line plot of cluster selection with the x-axis as the number of clusters, the left y-axis (solid pink line) as the silhouette score, and the right y-axis (dashed black line) as the median cluster size (quantified as number of genes). Input data for

clustering is glioblastoma (GBM) minus healthy intra-cellular gene-gene interaction scores and was completed via Ward’s method utilizing a criterion of “maxclust”.

b) Bar plot of each cluster from panel (a) of the differences between GBM and healthy intra-cellular gene-gene interaction matrices on the x-axis and the average difference in interaction score on the y-axis with positive indicating GBM-enrichment and negative indicating healthy-enrichment. Error bars represent standard error and bar heights represent mean.

c) Heatmap of the difference between GBM and healthy intra-cellular gene-gene interaction matrices, color represents difference with red representing positive for GBM-enriched, blue representing negative for healthy-enriched, and yellow for no difference. Clustering from panel (a) is labeled on the plot, legend on the right.

d) Bar plot of the pathways, x-axis, enriched for GBM or healthy enriched interaction difference clusters from panel (a) from the gene ontology (GO) biological processes (BP) database as quantified by $-\log_{10}$(p-value), y-axis and color, legend for y-axis and color is on the bottom.

Source data are provided as a Source Data file.

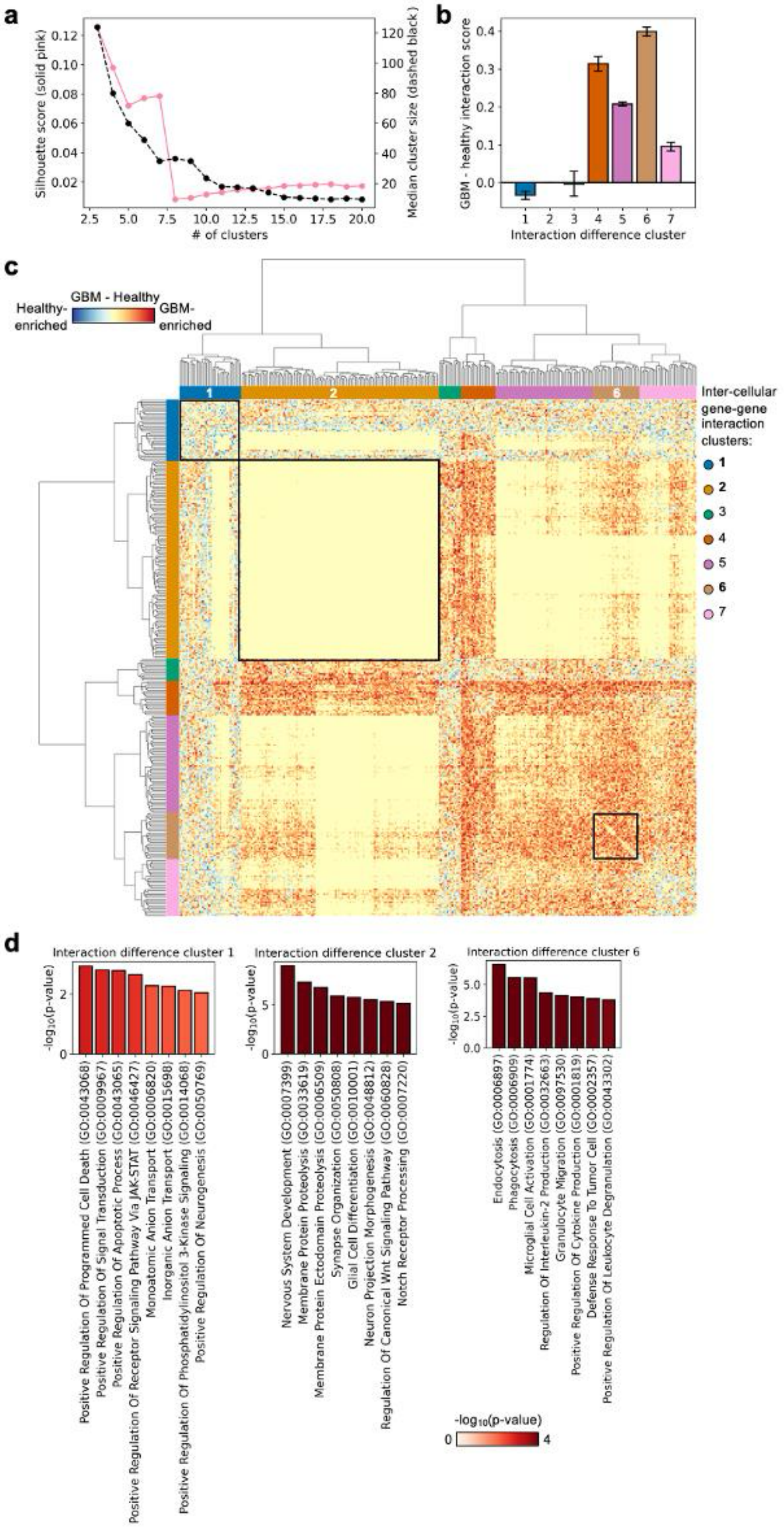

Supplementary Fig. 3 – comparison of inter-cellular gene-gene interactions between glioblastoma and healthy human brains:

a) Line plot of cluster selection with the x-axis as the number of clusters, the left y-axis (solid pink line) as the silhouette score, and the right y-axis (dashed black line)

as the median cluster size (quantified as number of genes). Input data for clustering is glioblastoma (GBM) minus healthy inter-cellular gene-gene interaction scores and was completed via Ward's method utilizing a criterion of "maxclust".

e) Bar plot of each cluster from panel (a) of the differences between GBM and healthy inter-cellular gene-gene interaction matrices on the x-axis and the average difference in interaction score on the y-axis with positive indicating GBM-enrichment and negative indicating healthy-enrichment. Error bars represent standard error and bar heights represent mean.

b) Heatmap of the difference between GBM and healthy inter-cellular gene-gene interaction matrices, color represents difference with red representing positive for GBM-enriched, blue representing negative for healthy-enriched, and yellow for no difference. Clustering from panel (a) is labeled on the plot, legend on the right.

c) Bar plot of the pathways, x-axis, enriched for GBM or healthy enriched interaction difference clusters from panel (a) from the gene ontology (GO) biological processes (BP) database as quantified by $-\log_{10}$(p-value), y-axis and color, legend for y-axis and color is on the bottom.

Source data are provided as a Source Data file.

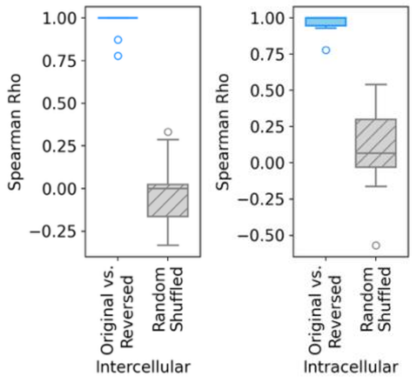

Supplementary Fig. 4 – Comparisons of Celcomen behavior for graphs within the same Markov equivalence class: Boxplots of the Spearman correlation coefficient (rho), y-axis, between Celcomen's predicted gene-gene interaction matrices from a given inter or intra -cellular graph and the same graph with all directed edges reversed (i.e. in the same Markov equivalence class). On the x-axis, the observed correlation is in blue on the left and the correlation between randomly shuffled values of the observed gene-gene interaction matrices is on the right in hatched gray. Box plots represent median and interquartile range. Source data are provided as a Source Data file.

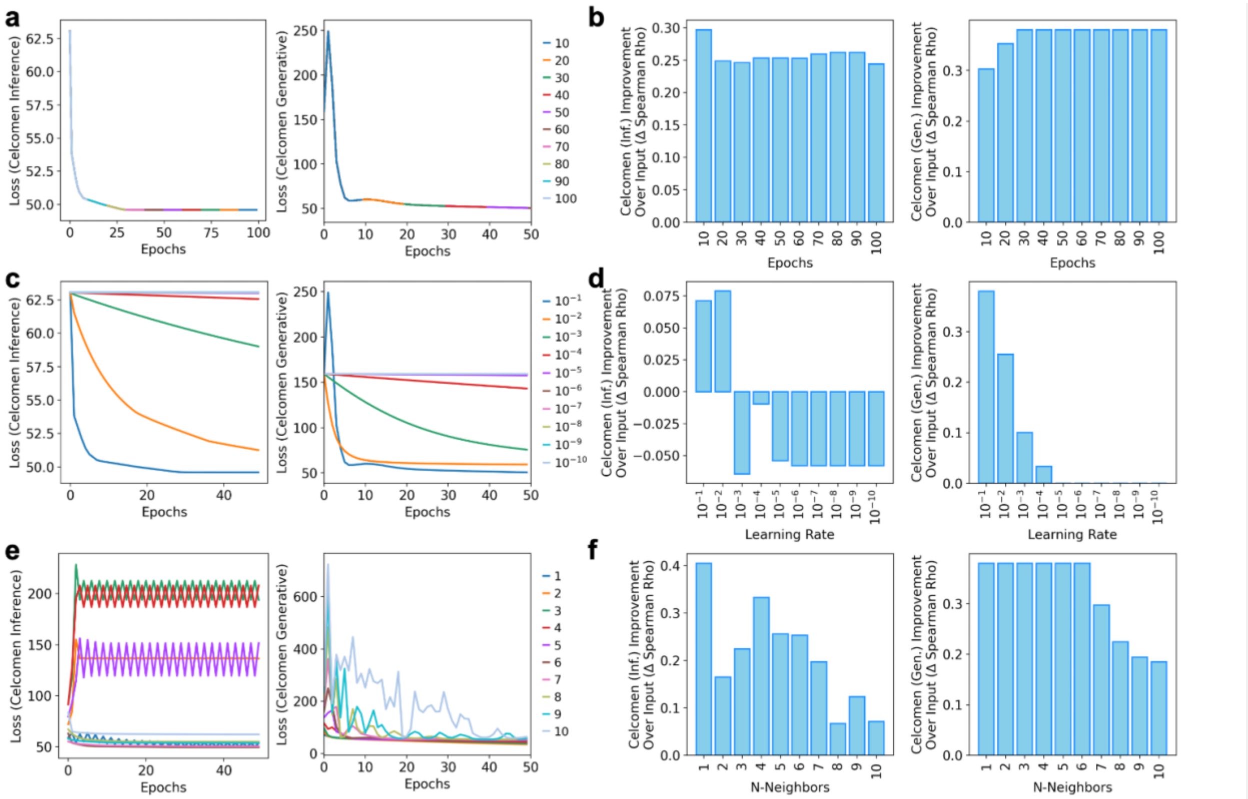

Supplementary Fig. 5 – Sensitivity analysis for number of epochs, learning rate and number of neighbors: Line plots (panels a, c, and e) represent loss curves for Celcomen's inference module (far left) and generative module (center left). Bar plots (panels b, d, and f) represent the Spearman correlation coefficient between the ground truth gene-gene interaction matrix and that inferred by Celcomen's inference module (center right) or from data generated by Celcomen's generative module (far right), y-axis is represented by deltas as we subtract the observed correlation between the ground truth and a random shuffling of the data (i.e. subtracting the background). In each row, we modulated a different parameter: number of epochs in the upper row (panels a and b), learning rate in the middle row (panels c and d), and n-neighbors considered in the bottom row (panels e and f). Source data are provided as a Source Data file.

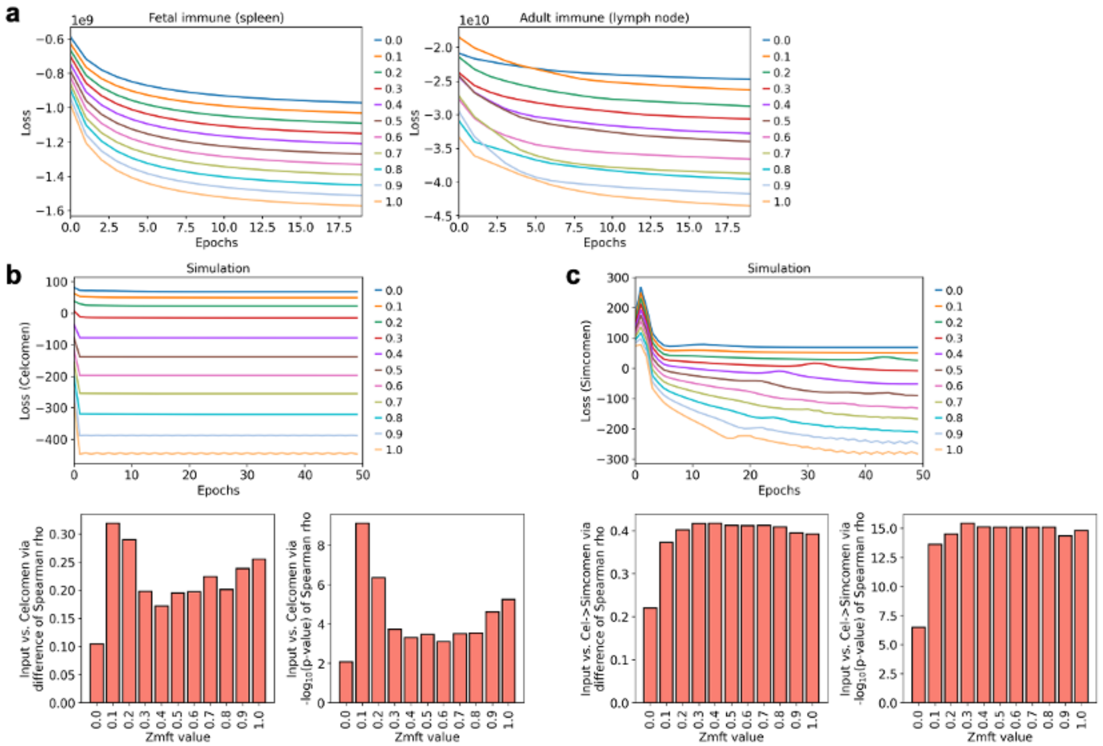

Supplementary Fig. 6 – Celcomen model performance as a function of *zmft_scalar:* Top (for panels (a), (b), and (c)): Line plot of Celcomen model loss, y-axis, throughout epochs of training, x-axis, with models trained on data with different *zmft_scalar* values, see color legend on the right. Bottom (for panels (b) and (c)): Bar plots of the differences (bottom left) or Mann-Whitney U-test p-values (bottom right) comparing the Spearman correlation coefficients, rho, of the ground truth with random raw input and with Celcomen's learned interactions, as a function of the *zmft_scalar* value, x-axis. Source data are provided as a Source Data file.

Supplemental Notes: Causal disentanglement for spatial perturbation modeling

1 Notation

- s_i^α, count values for spot/cell i and gene α,
- $\mathcal{H}$, Hamiltonian of a system,
- $Z = \sum_{\{s_i^\alpha\}} e^{\mathcal{H}(\{s_i^\alpha\})}$, the partition function,
- $\sum_{<i,j>nn}$, sum over pairs of nodes $\{i, j\}$ that are nearest neighbors,
- $g_{\alpha\beta}$, Lagrange multiplier enforcing gene-gene correlations,
- q, the number of nearest neighbors (that we assume are interacting),
- $\mathcal{S}$, the entropy functional,
- S, the number of spots/nodes in the spatial graph,
- N, the number of features/genes in the graph,
- J_{ij}, the spatial adjacency matrix between spots/nodes in the graph,
- $\langle\rangle_P$, the average with respect to the probability distribution P,
- $\langle\rangle_{\text{emp}}$, the empirical average with respect to the observed samples,
- $P(s_i^\alpha) \in L^1$, the probability density of the count matrix of a spatial, transcriptomics experiment equals the matrix s_i^α.

2 Motivation and Inspiration

Causal inference in machine learning aims to extract causal structures from observational data. As such, it stands in between correlation-based methods, and mechanistic models. Suppose for instance that biology imposes the co-localization of genes 1 and 2, and genes 2 and 3 in nearest neighbors. Since half of the time the nearest neighbor of a nearest neighbor is also a nearest neighbor, there will be (spurious) co-localization also of genes 1 and 3 in nearest neighbors. A causal model should be able to de-confound such spurious connections

within spatial correlations, even without mechanistic data such as epigenetic information.

Inspiration for our work comes from the notion of force in physics. In broad strokes, we aim to learn the "least" number of forces (i.e. causal mechanisms which force the co-localization of pairs of genes) that can explain the empirical spatial correlations of pairs of genes. "Least" here is meant in the sense of smallest entropy, not absolute number of forces (although we could additionally impose a L1 norm penalty on the force matrix).

In the Lagrangian formulation of classical physics, we think of time evolution of physical objects as an optimization problem (optimizing the action) such that certain constraints imposed by Lagrange multipliers are obeyed. One can show that Lagrange multipliers are equal to the force required to impose the corresponding constraint, which means that they are meaningful, physical quantities. At the same time, imposing the constrain via the Lagrange multipliers allows us to remain agnostic about the nature of the force (be it electromagnetism, gravity, or nuclear forces) that imposes the constraint. For an ant forced to walk on the surface of a table, this force (not letting it go through the table) happens to be electromagnetism, but we don't need to know this in advance to calculate its value.

Similarly in single cell genomics, measurements are valued in a high-dimensional gene expression space, but they often are hypothesised to lie on a much lower dimensional surface (see manifold hypothesis) due to biological mechanisms (already discovered or not) that "force" our measurements to lie on it. Uncovering such causal links is the first step to identifying the underlying molecular mechanisms. We use Lagrange multipliers to impose the empirical co-localization of genes. Since, Lagrange multipliers are meaningful and physical quantities, finding them is likely to be a well-posed problem, leading to causally identifiable models. Moreover, if we could make the weights of the trained network equal to the Lagrange multipliers of our problem, then recovering the network weights would be easier.

3 Model Assumptions

Our model is the *unique* model that follows from three assumptions:

- that our model's expected gene-gene correlations across nearest neighbors match exactly the empirical ones,
- that our model's expected gene-gene correlations within spots/cells match exactly the empirical ones,
- that any other variables influencing the gene expression can be sufficiently modelled as white noise.

These three assumptions can be summarized in the following equation for the entropy

$$\begin{aligned}\mathcal{S}(P(\{s_i^\alpha\}), g_{\alpha\beta}, g'_{\alpha\beta}) = &- \sum_{\{s_i^\alpha\}} P(\{s_i^\alpha\}) \log(P(\{s_i^\alpha\})) \\ &+ \sum_{\alpha,\beta} g_{\alpha\beta}(\langle \sum_{i,j\ nn} s_i^\alpha s_j^\beta \rangle_P - \langle \sum_{i,j\ nn} s_i^\alpha s_j^\beta \rangle_{\text{emp}}) \\ &+ \sum_{\alpha,\beta} g'_{\alpha\beta}(\langle \sum_i s_i^\alpha s_i^\beta \rangle_P - \langle \sum_i s_i^\alpha s_i^\beta \rangle_{\text{emp}}) \end{aligned} \quad (1)$$

where s_α^i is the spatial gene expression and $P(\{s_\alpha^i\})$ is the probability distribution over possible spatial transcriptomics samples, and $g'_{\alpha\beta}$, $g_{\alpha\beta}$ are Lagrange multipliers that enforce our assumptions 1 and 2.

Our task now is to maximize the entropy functional 1 over all possible functions $P \in L^1(\mathbb{R}^{N\times S})$ and matrices $g_{\alpha\beta}$ and $g'_{\alpha\beta}$,

$$\max_{P,g,g'} \mathcal{S}(P(\{s_i^\alpha\}), g_{\alpha\beta}, g'_{\alpha\beta}). \quad (2)$$

4 Model Derivation

We should note that the optimization problem above is a particularly hard non-parametric problem, since it requires optimizing over not some numerical parameters but over the space of normalised functions. Relatedly, the entropy is not a function over numbers, but a functional over functions. Using functional calculus, we perform the maximization of the entropy functional in eq. 1 over all functions $P \in L^1(\mathbb{R}^{N\times S})$, to arrive at a simpler optimization problem over g alone. This simpler optimization problem is more amenable by neural networks and will reveal the architecture our network should assume.

Proposition 1 (Extremization over P)**.** *The following two optimization problems are equivalent*

- *Maximizing the entropy functional in eq 1 over all possible functions $P \in L^1(\mathbb{R}^{N\times S})$ and matrices $g_{\alpha\beta}$ and $g'_{\alpha\beta}$*

$$\max_{P,g,g'} \mathcal{S}(P(\{s_i^\alpha\}), g_{\alpha\beta}, g'_{\alpha\beta}) \quad (3)$$

  where $\mathcal{S}$ is given by 1,

- *Minimizing the empirical log likelihood over matrices $g_{\alpha\beta}$ and $g'_{\alpha\beta}$*

$$\min_{g,g'} \langle \log P \rangle_{emp} = \min_{g,g'} \left(- \log Z(g_{\alpha\beta}) + g_{\alpha\beta} C_{\alpha\beta}^{emp} + g'_{\alpha\beta} C'^{\,emp}_{\alpha\beta} \right) \quad (4)$$

  where $C_{\alpha\beta} = \sum_{i,j} s_{j\alpha} J_{ji} s_{i\beta}$ and $C'_{\alpha\beta} = \sum_i s_{i\alpha} s_{i\beta}$.

Proof. Optimizing a functional requires taking derivatives with respect to functions. In particular, using $\frac{\delta \int f(x)dx}{\delta f(y)} = \delta(x-y)$, we can maximize S with respect to P:

$$0 = \frac{\delta \mathcal{S}}{\delta P(s')} = -\log P(\{s'^{i}_{\alpha}\}) - 1 + \sum_{\alpha,\beta} g_{\alpha\beta} \sum_{i,j\ nn} s'^{\alpha}_i s'^{\beta}_j + \sum_{\alpha,\beta} g'_{\alpha\beta} \sum_i s'^{\alpha}_i s'^{\beta}_i \quad (5)$$

$$\Rightarrow\ P(\{s^\alpha_i\}|\{g'_{\alpha\beta}, g_{\alpha\beta}\}) = \frac{e^{\mathcal{H}(\{s^\alpha_i\})}}{Z} \quad (6)$$

where we normalized the probability function and denote

$$\mathcal{H} = \sum_{\alpha\beta}\sum_i s^\alpha_i g'_{\alpha\beta} s^\beta_i + \sum_{\alpha\beta}\sum_{<i,j>nn} s^\alpha_i g_{\alpha\beta} s^\beta_j \quad (7)$$

$$= \sum_{\alpha\beta}\sum_i s^\alpha_i g'_{\alpha\beta} s^\beta_i + \sum_{\alpha\beta}\sum_{i,j} s^\alpha_i J_{ij} g_{\alpha\beta} s^\beta_j, \quad (8)$$

$$Z = \sum_{s^\alpha_i} e^{\mathcal{H}(\{s^\alpha_i\})}. \quad (9)$$

Maximizing with respect to the Lagrange multipliers $g_{\alpha\beta}$, $g'_{\alpha\beta}$ gives:

$$0 = \langle \sum_{i,j\ nn} s^\alpha_i s^\beta_j \rangle_P - \langle \sum_{i,j\ nn} s^\alpha_i s^\beta_j \rangle_{\text{emp}}\ , \quad (10)$$

$$0 = \langle \sum_i s^\alpha_i s^\beta_i \rangle_P - \langle \sum_i s^\alpha_i s^\beta_i \rangle_{\text{emp}}\ . \quad (11)$$

Moreover, by substituting 6 into 1 we get

$$\mathcal{S}(P(\{s^\alpha_i\}), g_{\alpha\beta}, g'_{\alpha\beta}) = \log Z - g_{\alpha\beta} \langle \sum_{i,j\ nn} s^\alpha_i s^\beta_j \rangle_{\text{emp}} - g'_{\alpha\beta} \langle \sum_i s^\alpha_i s^\beta_i \rangle_{\text{emp}} \quad (12)$$

$$= -\langle \log P(s) \rangle_{\text{emp}} \quad (13)$$

Therefore maximizing $\mathcal{S}$ is equivalent to minimizing

$$\langle \log P \rangle_{\text{emp}} = -\log Z(g_{\alpha\beta}) + g_{\alpha\beta} C^{\text{emp}}_{\alpha\beta} + g'_{\alpha\beta} C'^{\text{emp}}_{\alpha\beta} \quad (14)$$

where

$$C_{\alpha\beta} = \sum_{i,j} s_{j\alpha} J_{ji} s_{i\beta} = JSG'. \quad (15)$$

We now recognise this as the message passing equation for a Graph Convolutional Network (GCN) [2].

□

In summary, we have shown that our original constrained optimization problem is equivalent to an optimization problem over only the Lagrange multipliers

g, and g'. The loss function of this new optimization problem is eq 14, where J is the adjacency matrix of the graph, g' are the intercellular Lagrange multipliers, and s is the gene expression matrix (cell by gene). We now notice that eq 15 can be interpreted as the message passing term (see equation 8 in [2]) where J is the adjacency matrix of a GNN, S is the feature matrix (node by feature) of the graph data, and G' are the weights of one GNN layer.

In other words, Proposition 1 shows that the new optimization problem is the optimization problem of a (k-hop) GNN [3] with a new and simpler loss function, and after the GNN has been trained/optimized its weights will be equal to the values of the Lagrange multipliers of the original problem.

5 Mean Gene Approximation

Despite the simpler loss function of our k-hop GCN, it is still intractable to compute, because calculating the partition function (and its derivatives) requires summing over a large number of possible spatial transcriptomics datasets.
Several famous algorithms in machine learning circumvent computing the partition function in different ways. For instance, a contrastive learning approach essentially takes the ratio of probabilities, thereby cancelling out the partition function; optimization approaches cast the avoid the computation of the partition by considering maximum a-posteriori estimator [1]; and, score-based diffusion [4] uses score-matching to learn a model of the gradient of the log of the probability density function, which again avoids computing the partition function completely.
In this paper, we introduce a novel approximation to the partition function, inspired from physics, which has not been used before in spatial transcriptomics to our knowledge. This is a new Mean Field Theory approximation

$$s_k^\alpha = \bar{s}_k^\alpha + \delta s_k^\alpha = m^\alpha + \delta s_k^\alpha \tag{16}$$

where we assume that the gene expression does not fluctuate much around the mean.

Using this, we can rewrite the exponent as

$$\begin{aligned}
s_i^\alpha g_{\alpha\beta} s_j^\beta &= g_{\alpha\beta}(\bar{s}_i^\alpha + \delta s_i^\alpha)(\bar{s}_j^\beta + \delta s_j^\beta) &(17)\\
&\approx g_{\alpha\beta}(\bar{s}_i^\alpha \bar{s}_j^\beta + \bar{s}_j^\beta \delta s_i^\alpha + \bar{s}_i^\alpha \delta s_j^\beta) &(18)\\
&= g_{\alpha\beta}(m^\alpha m^\beta + m^\beta (s_i^\alpha - m^\alpha) + m^\alpha (s_j^\beta - m^\beta)) &(19)\\
&= g_{\alpha\beta}(-m^\alpha m^\beta + m^\beta s_i^\alpha + m^\alpha s_j^\beta)\ . &(20)
\end{aligned}$$

where in the second line we used the MFT approximation to neglect terms of order higher than 2, and

$$s_i^\alpha g'_{\alpha\beta} s_i^\beta = g'_{\alpha\beta}(-m^\alpha m^\beta + m^\beta s_i^\alpha + m^\alpha s_i^\beta)\ . \tag{21}$$

This implies that the inter-cellular term in the exponent can be rewritten as

$$\sum_{\langle i,j\rangle}\sum_{\alpha,\beta} g_{\alpha\beta}(-m^\alpha m^\beta + m^\beta s_i^\alpha + m^\alpha s_j^\beta) = \frac{q}{2}\sum_i\sum_{\alpha,\beta} g_{\alpha\beta}(-m^\alpha m^\beta + m^\beta s_i^\alpha + m^\alpha s_i^\beta) \tag{22}$$

where q is the number of nearest neighbors that we assume are interacting, and therefore

$$\mathcal{H} = \frac{q}{2}\sum_i\sum_{\alpha,\beta} g_{\alpha\beta}(-m^\alpha m^\beta + m^\beta s_i^\alpha + m^\alpha s_i^\beta) \tag{23}$$

$$+\sum_i\sum_{\alpha,\beta} g'_{\alpha\beta}(-m^\alpha m^\beta + m^\beta s_i^\alpha + m^\alpha s_i^\beta) \tag{24}$$

$$=\sum_i\sum_{\alpha,\beta} \big(g'_{\alpha\beta} + \frac{q}{2}g_{\alpha\beta}\big)(-m^\alpha m^\beta + m^\beta s_i^\alpha + m^\alpha s_i^\beta) \tag{25}$$

since $g_{\alpha,\beta}$ is symmetric.

Lemma 1. *The following sum can be simplified as follows*

$$\sum_{\{s_i^\alpha\}} emp\Big[\sum_i\sum_{\alpha,\beta}(\frac{q}{2}g_{\alpha\beta})(m^\beta s_i^\alpha + m^\alpha s_i^\beta)\Big] = V_{\mathbb{S}^{n-1}}\left(\frac{e^{qH/2}-e^{-qH/2}}{qH/2}\right)^S \tag{26}$$

where S is the number of spots, $H_{\alpha\beta} = g_{\alpha\beta}+g_{\beta\alpha}$, $H = \sqrt{\sum_\beta(\sum_\alpha H_{\alpha\beta}m^\alpha)^2}$.

Proof.

$$Z = \sum_{\{s_i^\alpha\}} \text{emp}\left[\frac{q}{2}\sum_i \sum_{\alpha,\beta} g_{\alpha\beta}(m^\beta s_i^\alpha + m^\alpha s_i^\beta)\right] \tag{27}$$

$$= \sum_{\{s_i^\alpha\}} \text{emp}\left[\frac{q}{2}\sum_i \sum_{\alpha,\beta} (g_{\beta\alpha} m^\alpha s_i^\beta + g_{\alpha\beta} m^\alpha s_i^\beta)\right] \tag{28}$$

$$= \sum_{\{s_i^\alpha\}} \text{emp}\left[\frac{q}{2}\sum_i \sum_{\alpha,\beta} (g_{\beta\alpha} + g_{\alpha\beta}) m^\alpha s_i^\beta\right] \tag{29}$$

$$= \sum_{\{s_i^\alpha\}} \text{emp}\left[\frac{q}{2}\sum_i \sum_{\alpha,\beta} H_{\alpha\beta} m^\alpha s_i^\beta\right] \tag{30}$$

$$= \prod_i \left(\int_{s_i \in \mathbb{S}^n} ds_i\right) \text{emp}\left[\frac{q}{2}\sum_i \sum_{\alpha,\beta} H_{\alpha\beta} m^\alpha s_i^\beta\right] \tag{31}$$

$$= \prod_i \left(\int_{s_i \in \mathbb{S}^n} \text{emp}\left[\frac{q}{2}\sum_i H s_i^1\right] ds_i\right) \tag{32}$$

$$= V_{\mathbb{S}^{n-1}} \prod_i \left(\int_0^\pi \text{emp}\left[\frac{q}{2}\sum_i H\cos\theta\right] \sin\theta d\theta\right) \tag{33}$$

$$= V_{\mathbb{S}^{n-1}} \prod_i \left(\int_{-1}^1 \text{emp}\left[\frac{q}{2}\sum_i H u\right] du\right) \tag{34}$$

$$= V_{\mathbb{S}^{n-1}} \left(\frac{e^{qH/2} - e^{-qH/2}}{qH/2}\right)^S \tag{35}$$

$$\tag{36}$$

where S is the number of spots, $H_{\alpha\beta} = g_{\alpha\beta} + g_{\beta\alpha}$, $H = \sqrt{\sum_\beta(\sum_\alpha H_{\alpha\beta} m^\alpha)^2}$, and without loss of generality we assumed that the vector $\sum_\alpha H_{\alpha\beta} m^\alpha$ lies only along the first dimension. □

Now applying this proposition to our formula for the partition function, where we need to replace $g_{\alpha\beta} \to g_{\alpha\beta} + \frac{2}{q} g'_{\alpha\beta}$, gives

$$\log Z = -S \sum_{\alpha,\beta} \left(g'_{\alpha\beta} + \frac{q}{2} g_{\alpha\beta}\right) m^\alpha m^\beta$$
$$+ \log V_{\mathbb{S}^{n-1}}$$
$$+ S \log \frac{e^{H'/2} - e^{-H'/2}}{H'/2} \tag{37}$$

where S is the number of spots, $H'_{\alpha\beta} = q g_{\alpha\beta} + q g_{\beta\alpha} + 2g'_{\alpha\beta} + 2g'_{\beta\alpha}$, $H' = \sqrt{\sum_\beta(\sum_\alpha H'_{\alpha\beta} m^\alpha)^2}$

Using our 13, 4, 37, we have a complete formula for calculating the partition function and the only optimization remaining is over the Lagrange multipliers.

$$0 = \frac{\delta P(\{s_i^\alpha\})}{\delta g_{\alpha\beta}} \tag{38}$$

$$0 = \frac{\delta P(\{s_i^\alpha\})}{\delta g'_{\alpha\beta}} \tag{39}$$

In other words we want to look for the forces that are causing the empirical spatial gene expression. Since the Lagrange multipliers/forces are meaningful physical variables, they naturally equip our model with identifiability as we show in the next section.

6 Celcomen Architecture

To construct Celcomen, we used the Python packages of "PyG" (PyTorch Geometric), "PyTorch" and "scikit-learn". Celcomen uses two types of input: one encodes the graph structure, in the form of an adjacency matrix J for the k-NN graph created from the spatial locations of the cells and the hyperparameter n-neighbors; while the second is the log-transformed and then spherically normalized gene expression (such that the L_2 norm of the gene expression vector of each cell is equal to 1). The hyperparameter n-neighbors should be chosen based on what information we want to disentangle in our network. By default, it is set to 6, which means that Celcomen tries to disentangle the intra-cellular gene regulation from the regulation of the cell's gene expression by its 6 nearest neighbors.

Celcomen consists of one GCN layer (from the PyG implementation) with weights G' and both input and output dimensions equal to the number of genes; and one linear layer from the PyTorch implementation with weights G and where again both the input and output dimensions are equal to the number of genes. Celcomen uses these two layers to construct the node embedding of node n as follows

$$Z = JSG' + SG \tag{40}$$

and the loss function is calculated by

$$loss = log(\text{z-mft}) - \text{scalar} \times Tr(ZS^T) \tag{41}$$

where z-mft is the mean-field approximation derived in eq 37, and "scalar" is a hyperparameter that adjusts the weighting of the mean-field approximation to ensure the loss function is lower bounded even when the mean-field approximation assumption is violated in the data. This hyperparameter should be chosen between 0 (big adjustment) and 1 (no adjustment, i.e. the mean field approximation is exact and not an approximation) such that the training process stably converges to a minimum.

7 Identifiability

An important question we want to address is the identifiability of our model, i.e. whether there is a unique setting of the forces that leads to the empirical correlations in the data. If the identifiability property holds then our model will naturally be robust and causal in the sense that it can de-confound spurious correlations and recover almost the Markov equivalence class of the causality diagram, which is the best any method without interventional data can do.

In mathematical terminology, we want to determine whether there is some gauge symmetry that allows different sets of parameters to produce the same probability distribution.

Proposition 2 (Identifiability)**.** *The model defined by equation 6 is identifiable in the sense that*

$$\forall\{s_i^\alpha\}:\ P(\{s_i^\alpha\}|\{g_{\alpha\beta},g'_{\alpha\beta}\}) = P(\{s_i^\alpha\}|\{h_{\alpha\beta},h'_{\alpha\beta}\}) \tag{42}$$

$$\Rightarrow\ g_{\alpha\beta} = h_{\alpha\beta} and\, g'_{\alpha\beta} = h'_{\alpha\beta} \tag{43}$$

Proof. Let's pick i to be a cell/node that has at least one neighbor. If there is not such a cell then there wouldn't be a cell communication problem to model.

$$P(\{s_i^\alpha\}|\{g_{\alpha\beta},g'_{\alpha\beta}\}) = P(\{s_i^\alpha\}|\{h_{\alpha\beta},h'_{\alpha\beta}\}) \tag{44}$$

$$\Rightarrow \frac{dP(\{s_i^\alpha\}|\{g_{\alpha\beta},g'_{\alpha\beta}\})}{ds_i^\alpha} = \frac{dP(\{s_i^\alpha\}|\{h_{\alpha\beta},h'_{\alpha\beta}\})}{ds_i^\alpha} \tag{45}$$

Then we pick j to be any of the neighbors of cell i,

$$\frac{d^2P(\{s_i^\alpha\}|\{g_{\alpha\beta},g'_{\alpha\beta}\})}{ds_j^\beta ds_i^\alpha} = \frac{dP(\{s_i^\alpha\}|\{h_{\alpha\beta},h'_{\alpha\beta}\})}{ds_j^\beta ds_i^\alpha} \tag{46}$$

$$\Rightarrow \frac{d^2P(\{s_i^\alpha\}|\{g_{\alpha\beta},g'_{\alpha\beta}\})}{ds_j^\beta ds_i^\alpha}\bigg|_{s^\beta_{nn\ j}=0,s_i^\alpha=0} = \frac{dP(\{s_i^\alpha\}|\{h_{\alpha\beta},h'_{\alpha\beta}\})}{ds_j^\beta ds_i^\alpha}\bigg|_{s^\beta_{nn\ j}=0,s_i^\alpha=0} \tag{47}$$

$$\Rightarrow g_{\alpha\beta} = h_{\alpha\beta} \tag{48}$$

Alternatively, taking the second derivative with respect to the same cell i,

$$\frac{d^2P(\{s_i^\alpha\}|\{g_{\alpha\beta},g'_{\alpha\beta}\})}{ds_i^\beta ds_i^\alpha} = \frac{dP(\{s_i^\alpha\}|\{h_{\alpha\beta},h'_{\alpha\beta}\})}{ds_i^\beta ds_i^\alpha} \tag{49}$$

$$\Rightarrow \frac{d^2P(\{s_i^\alpha\}|\{g_{\alpha\beta},g'_{\alpha\beta}\})}{ds_i^\beta ds_i^\alpha}\bigg|_{s_i^\beta=0,s_i^\alpha=0} = \frac{dP(\{s_i^\alpha\}|\{h_{\alpha\beta},h'_{\alpha\beta}\})}{ds_i^\beta ds_i^\alpha}\bigg|_{s_i^\beta=0,s_i^\alpha=0} \tag{50}$$

$$\Rightarrow g'_{\alpha\beta} = h'_{\alpha\beta} \tag{51}$$

□

8 Simcomen (SCE): Generation Module

Our model offers a mathematically robust way of learning the distribution of spatial transcriptomics samples such that there is a 1-1 correspondence between a configuration of forces and the learned distribution of spatial transcriptomics samples.

Generating new samples from the learned distribution is classic problem that can be addressed for instance by Markov Chain Monte Carlo Methods. However, given the high dimensionality of the space of spatial transcriptomics, MCMC can be very computationally expensive. Therefore in our generation module, called Simulated Communication Energy (Simcomen, SCE), we produce new samples in a denoising like approach by fixing the parameters of our model and optimizing the probability of the given data, either sampled from a different distribution, e.g. having Gaussian represent noise, or to generate counterfactual samples, e.g. we intervene on a spot or cell and from that starting point we find the most likely spatial distribution of gene expression values under the learned distribution.

9 One-gene inter-cellular communication leads to a convex optimization problem

In this section we study the optimization problem of Z when there is only one gene. The main results of this section is the following theorem.

Lemma 2 (MFT approximation leads to convex optimization problem)**.** *When the target space is of dimension one (i.e. there is only one feature), the minimization problem of* $\min_{g_{\alpha\beta}} \langle \log P \rangle_{emp}$*, where the partition function is approximated by MFT (see eq. 37), is a convex problem.*

Proof. For one gene we have

$$H = \sqrt{\sum_\beta (\sum_\alpha H_{\alpha\beta} m^\alpha)^2} = |H_{11} m| = 2|g_{11} m| \tag{52}$$

and therefore

$$\log Z(g_{11}) = -\frac{q}{2} S g_{11} m^2 + \log V_{\mathbb{S}^{n-1}} + S \log \frac{e^{q|g_{11}m|} - e^{-q|g_{11}m|}}{q|g_{11}m|} \tag{53}$$

$$\Rightarrow \langle \log P\rangle_{\text{emp}} = -\log Z(g_{11}) + g_{11}C_{11}^{\text{emp}} \quad (54)$$

$$= \frac{q}{2}Sg_{11}m^2 - \log V_{\mathbb{S}^{n-1}} - S\log\frac{e^{q|g_{11}m|} - e^{-q|g_{11}m|}}{q|g_{11}m|} + g_{11}C_{11}^{\text{emp}} \quad (55)$$

$$\Rightarrow \frac{d}{dg_{11}}\langle \log P\rangle_{\text{emp}} = \frac{q}{2}Sm^2 - S\left(\frac{q|m|(\pm 1)}{\tanh(q|g_{11}m|)} - \frac{(\pm 1)}{|g_{11}|}\right) + C_{11}^{\text{emp}} \quad (56)$$

$$\Rightarrow \frac{d}{dg_{11}}\langle \log P\rangle_{\text{emp}} = \frac{q}{2}Sm^2 - S\left(\frac{q|m|}{\tanh(q|m|g_{11})} - \frac{1}{g_{11}}\right) + C_{11}^{\text{emp}} \quad (57)$$

where the + sign is for $g_{11} > 0$ and the minus sign for $g_{11} < 0$.
As we can see in the figure below, $-Sqm \leq -S\left(\frac{q|m|}{\tanh(q|m|g_{11})} - \frac{1}{g_{11}}\right) \leq Sqm$ eq. 57 has one root only if $\frac{m}{2} < 1$, otherwise it is always positive. Another necessary constraint similarly exists for C_{11}^{emp} which tells us that the mean field theory approximation might sometimes need to be adjusted. Another direct prediction from equation 57 is that there is at most one root, and therefore our optimization problem is convex.

□

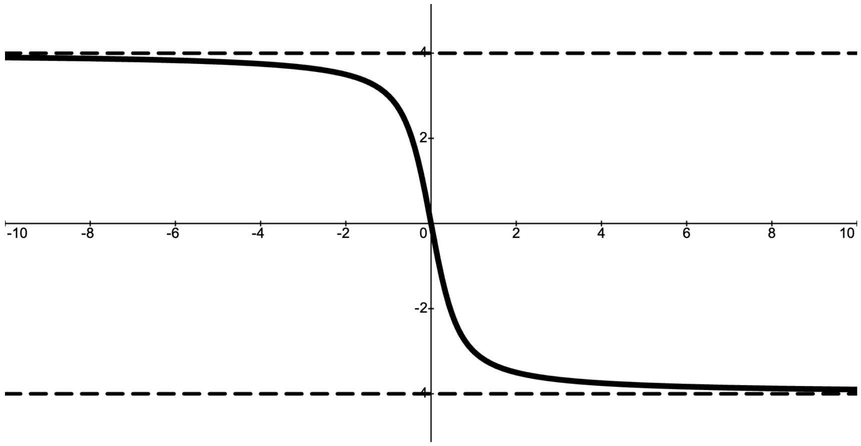

The second derivative is

$$\Rightarrow \frac{d^2}{d(g_{11})^2}\langle \log P\rangle_{\text{emp}} = -S\left(-q^2|m|^2\frac{1}{\sinh^2(q|m|g_{11})} + \frac{1}{(g_{11})^2}\right) \quad (58)$$

Supplementary References

[1] Eldad Haber, Moshe Eliasof, and Luis Tenorio. "Estimating a potential without the agony of the partition function". In: *SIAM Journal on Mathematics of Data Science* 5.4 (2023), pp. 1005–1027.

[2] Thomas N. Kipf and Max Welling. "Semi-Supervised Classification with Graph Convolutional Networks". In: *International Conference on Learning Representations*. 2017. URL: https://openreview.net/forum?id=SJU4ayYgl.

[3] Giannis Nikolentzos, George Dasoulas, and Michalis Vazirgiannis. *k-hop Graph Neural Networks*. 2020. arXiv: 1907.06051 [stat.ML]. URL: https://arxiv.org/abs/1907.06051.

[4] Yang Song et al. *Score-Based Generative Modeling through Stochastic Differential Equations*. 2021. arXiv: 2011.13456 [cs.LG]. URL: https://arxiv.org/abs/2011.13456.
